# Factor analysis of the Resilience Scale for Brazilian caregivers of people with Alzheimer’s disease

**DOI:** 10.47626/2237-6089-2020-0179

**Published:** 2021-12-10

**Authors:** Alexandre Magno Frota Monteiro, José Pedro Simões, Raquel Luiza Santos, Nathália Kimura, Maria Alice Tourinho Baptista, Marcia Cristina Nascimento Dourado

**Affiliations:** 1 Centro para Doença de Alzheimer e Outros Transtornos Mentais da Velhice Instituto de Psiquiatria Universidade Federal do Rio de Janeiro Rio de Janeiro RJ Brazil Centro para Doença de Alzheimer e Outros Transtornos Mentais da Velhice, Instituto de Psiquiatria, Universidade Federal do Rio de Janeiro (UFRJ), Rio de Janeiro, RJ, Brazil.; 2 Departamento de Sociologia e Ciência Política Universidade Federal de Santa Catarina Florianópolis SC Brazil Departamento de Sociologia e Ciência Política, Universidade Federal de Santa Catarina (UFSC), Florianópolis, SC, Brazil.

**Keywords:** Resilience, dementia, factorial analysis, caregiver, validity

## Abstract

**Introduction:**

Resilience is a dynamic process that acts to modify the effects of an adverse life event. In this study, we aimed to test the construct validity of the Resilience Scale by employing exploratory and confirmatory procedures, and to investigate the relationship between caregiver’s resilience and clinical status of people with Alzheimer’s disease.

**Methods:**

A sample of 143 dyads of people with Alzheimer’s disease and their primary caregivers were included.

**Results:**

The total Resilience Scale mean score was 140.3 (standard deviation [SD] = 16.289), ranging from 25 to 175, indicating a high level of resilience. Cronbach’s alpha was high (α = 0.77), indicating excellent internal consistency. The mean of corrected item-total correlation coefﬁcients was moderate. The Resilience Scale presented a four-factor solution with a well-deﬁned structure: sense of life and self-sufficiency, perseverance, self-confidence and equanimity, and meaningfulness.

**Conclusion:**

The findings indicate excellent internal consistency of the Resilience Scale when used to evaluate psychological and emotional difficulties of caregivers, even though the correlations observed between the Resilience Scale and clinical variables were not significant for functionality, mood, awareness, neuropsychiatric symptoms, or burden.

## Introduction

Resilience may be defined as a dynamic process involving the interaction between both risk and protective factors, both internal and external to the individual, that act to modify the effects of an adverse life event.^1,2^ Risk factors are individual or environmental obstacles that would increase an individual’s vulnerability to negative outcomes, such as the development of physical and mental illness or non-effective coping.^3^ Protective factors are characteristics that reduce or prevent the occurrence of problems and result in positive and adaptive results.^4^ Consequently, resilience does not involve invulnerability to stress, but, rather, the ability to recover from negative events.^5^

Resilience is a construct that is cognitive in nature, present in all people, differing only in its level.^3^ Since it is presented differently from subject to subject, there is an interest in developing methods of evaluating resilience, seeking to prevent diseases and promote mental health. In general, to identify whether an individual is resilient or not, two evaluations are required.^1,2^ First, the individual should be threatened by a high-risk state, or exposed to severe adversity or trauma.^6^ Second, the quality of the individual’s adaptation to the adverse life event or development should be good.^6^ Good adaptation may be operationally defined through indicators associated with functional competency in specific developmental domains, which imply behavioral achievements expected in specific areas.^7^

In dementia research, the stress and burden associated with caregiving, which impacts the caregivers’ physical health and increases their mortality risk, is well known.^8^ Caregivers of people with Alzheimer’s disease (AD) have been shown to present more stress, burden, and depression compared to caregivers of people with other diseases.^4^ However, despite the negative aspects involving the care of people with AD, many caregivers report a variety of positive experiences related to caregiving.^2^ The theoretical resilience framework developed by Windle & Bennett^3^ recognizes that caregivers will draw on individual resources, but also interact with their environment by employing community and societal resources which may facilitate or hinder resilience.^3^ The absence of resources may lead to poor outcomes or further caring challenges. Considering this framework, resilience can be described as “the process of negotiating, managing, and adapting to significant sources of stress or trauma.”^3^ Assets and resources within the individual, their life, and environment facilitate this capacity for adapting and “bouncing back” in the face of adversity.^3^

A gap in the literature about the resilience of caregivers of people with AD still deserves attention. It is well known that neuropsychiatric symptoms, impaired awareness of disease, and deficits in functionality are related to increased burden and decreased quality of life (QoL) of the caregiver.^9^ Nevertheless, few studies consider the influence of these clinical symptoms on the level of caregiver’s resilience.^10^ Therefore, in dementia research, it is important to assess people with AD-caregiver dyads to evaluate whether resilience is an individual characteristic or whether it is related to the level of severity of dementia symptoms. Rosa et al.^10^ found no significant difference in resilience between caregivers of mild and moderate people with AD, but upon analyzing the factors related to resilience in both groups, the results suggested that caregivers’ resilience is driven by different factors according to disease severity.

Understanding the resilience of caregivers of people with AD is crucial to the development of intervention strategies that can contribute to the improvement of their emotional disorders, such as anxiety, stress, and depression.^5^ Studies indicate that the general condition of the caregiver can interfere with the quality of care provided to people with AD, and may even lead to neglect or abuse of the elderly, as well as to their early institutionalization.^5^ Caregivers with higher levels of resilience may cope more effectively during stressful times. Conversely, for caregivers who have lower levels of resilience and limited coping mechanisms to fulfill the caregiving demands, external resources need to be provided, as it is known that the surrounding environment can affect the individual’s strength.^5^

One way of ensuring data quality in resilience research is to only use measures that have been validated. Currently, there is no standardized measure of caregiver’s resilience in dementia. The few studies that attempted to investigate resilience among caregivers of people with AD have indicated that the most frequent assessment instruments employed were the complete and brief versions of the Resilience Scale developed by Wagnild & Young.^1^

The purpose of the Resilience Scale is to identify the degree of individual resilience, considered a positive personality characteristic that enhances individual adaptation.^1^ The original validation of the scale identified five interrelated components that constitute resilience: equanimity (a balanced perspective of one’s life experiences); perseverance (the act of persistence despite adversity or discouragement); self-reliance (a belief in oneself and one’s capabilities); meaningfulness (the realization that life has a purpose and the valuation of one’s contributions); and existential aloneness (the realization that each person’s life path is unique).^1,4^

The determinant factors of resilience were classified, according to the validated Resilience Scale, into three areas. The first area was psychological, i.e., higher levels of resilience were associated with positive cognitions,^7^ resources,^7^ optimism,^8^ self-efficacy,^8^ internal locus of control,^8^ full engagement in daily activities^11^ and search for challenges.^11^ There were also associations with decreased burden,^11,12^ stress,^7^ neuroticism,^13^ perceived control,^14^ and distress.^14,15^ The second area was biological, meaning that higher levels of resilience were associated with less depression^13^ and better physical health.^13^ Finally, there was the social area,^16^ in which higher levels of resilience were associated with social factors such as good social support,^13^ satisfaction with social support^11^ and individual, family, and community resources.^17^

The Resilience Scale was adapted to Brazilian Portuguese and has had its construct validity evaluated by Pesce et al.,^18^ using a sample of students from public schools in Rio de Janeiro, Brazil. The Brazilian Portuguese Resilience Scale showed good semantic equivalence for general meaning (above 90.0%) and referential meaning (above 85.0%). Chronbach’s alphas were 0.85 in the pilot study and 0.80 in the total sample. Kappa between the two points in time was regular and moderate, and the intraclass correlation coefficient was 0.746 (p = 0.000). Factorial exploratory analysis indicated three non-homogeneous factors. Construct validity demonstrated a direct and significant correlation with self-esteem, family supervision, life satisfaction, and social support.^18^ However, one shortcoming of the study by Pesce et al.^18^ is that construct validity was investigated focusing on students. Therefore, a reliable measure of the resilience of caregivers of people with AD is still necessary.

The objectives of this study were to test the construct validity of the Resilience Scale employing exploratory and confirmatory procedures and to investigate the relationship between caregiver’s resilience and clinical status of the person with AD.

## Material and methods

### Study design

This was a cross-sectional study.

### Participants

A consecutive series of people with AD and their caregivers (n = 143 dyads) were recruited from an outpatient clinic in Rio de Janeiro, Brazil. People with AD were diagnosed with AD according to the Diagnostic and Statistical Manual of Mental Disorders, 5th edition (DSM-5).^19^ Sample size was calculated based on psychometric criteria that suggested the inclusion of 5 to 10 participants per item, according to Treiblmaier & Filzmoser.^20^

People with mild to moderate AD according to the Clinical Dementia Rating (CDR)^21^ and scores ranging from 13 to 26 in the Mini-Mental State Examination (MMSE)^22^ were included in the study. Exclusion criteria for people with AD were the presence of psychiatric or neurological disorders, such as aphasia, head trauma, alcohol abuse, and epilepsy, as deﬁned by the DSM-5^19^ criteria.

For caregivers, the following inclusion criteria were taken into consideration: 1) having had at least one year of formal education; 2) having contact with the care recipient at least three times a week; and 3) MMSE ≥ 28. We excluded caregivers with a history of neuropsychiatric or cognitive disorders and those who did not meet the care recipient at least three times a week. All caregivers in the study were identified as primary or secondary caregivers, thus being able to provide detailed information about the people with AD they cared for.

Measures of cognition,^22^ awareness of disease,^23^ and depression^24,25^ were administered to people with AD. Caregivers were assessed with measures of resilience^18^ and burden.^26^ Also, they were asked to provide information about the people with AD on the following topics: demographics, awareness of disease,^23^ mood,^24,25^ neuropsychiatric symptoms,^27^ and dementia severity.^21^

The dyad was interviewed separately, simultaneously, face to face, by trained neuropsychologists for approximately 90 minutes at the outpatient Alzheimer’s Disease Center (Centro para Doença de Alzheimer e outros Transtornos Mentais da Velhice, Universidade Federal do Rio de Janeiro), and the assessment instruments were presented in the same order to all participants, to ensure standardization. All assessments were completed in a single session.

This study was approved by the ethics committee of the Instituto de Psiquiatria, Universidade Federal do Rio de Janeiro. The dyads signed informed consent terms before the evaluation. All the procedures in the present study were conducted in accordance with the ethical standards of the relevant national and institutional committees on human experimentation and the Declaration of Helsinki.

### Instruments

#### Caregivers’ measurements

**Resilience.** The Resilience Scale consists of 25 items that measure levels of positive adaptation in the face of adverse life events. The scale captures the resilience construct, serenity, perseverance, self-confidence, sense of life, and self-sufficiency. Scores range from 25-175, with higher values indicating greater resilience. Scores below 125 indicate low resilience, between 125-145 medium resilience, and above 145 high resilience.^18^

**Cognition.** The MMSE consists of 30 items that assess orientation, learning, short-term memory, language, comprehension, and basic motor skills. The total score ranges from 0-30, with lower scores indicating more impaired cognition.^22^

**Burden.** The Zarit Burden Interview (ZBI) was used to assess caregiver burden. ZBI comprises 22 items designed to measure the impact of caring for someone with dementia on the caregiver’s life. Total scores range from 0-88. Higher scores indicate a higher level of burden.^26^

#### Caregivers’ measurements about people with AD

**Neuropsychiatric symptoms.** The Neuropsychiatric Inventory (NPI) was used to assess for common neuropsychiatric symptoms reported in dementia. Total scores range from 0-144. Higher scores indicate greater levels of neuropsychiatric symptoms.^27^

**Severity of dementia.** The Clinical Dementia Rating (CDR) scale evaluates possible stages of cognition and function, with the following scores: 0 (no dementia), 0.5 (questionable dementia), 1 (mild dementia), 2 (moderate dementia) and 3 (severe dementia).^21,28^

**Functionality.** The Pfeffer Functional Activities Questionnaire (FAQ) evaluates functional abilities. The ratings for each item range from normal (0) to dependent (3). Scores may range from 0 to 30, with higher scores indicating worse functional status.^29^

#### Measurement used in people with AD

**Cognition.** MMSE, as explained above.

**Mood.** The Cornell Scale for Depression in Dementia (CSDD) was used to assess mood symptoms, physical signs, circadian functions, and behavioral symptoms related to depression among people with dementia. Scores above 13 indicate the presence of depression.^24,25^

**Awareness of the disease.** The Assessment Scale of Psychosocial Impact of the Diagnosis of Dementia (ASPIDD) is a 30-question scale designed to evaluate awareness of disease across four domains, including awareness of cognitive functioning and health condition, awareness of functional impairments, awareness of emotional state, and awareness of social functioning and relationships. Scores are based on the degree of discrepancy between the responses of the dyad, with one point being scored for each discrepant response. The ratings of awareness range from preserved (0-4), mildly impaired (5-11), moderately impaired (12-17), to absent (over 18).^23^

## Data analysis

All analyses were performed using the Statistical Package for the Social Sciences (SPSS; SPSS Inc., Chicago, IL, USA). The Kaiser-Meyer-Olkin (KMO) test was conducted and the measure was calculated to evaluate sampling adequacy in order to carry out an exploratory factor analysis. A KMO value close to 1 indicates that the sum of partial correlations is not large relative to the sum of correlations, and so factor analysis should yield distinct and reliable factors. Revelle et al.^30^ suggested accepting a value > 0.5 and recommended a minimum of 5 observations per variable.^31^

We used maximum likelihood extraction instead of principal component analysis because the latter procedure does not discriminate between shared and unique variance, inﬂating variance estimates.^32^ Maximum likelihood has been recommended as an extraction method even in cases when data is not normally distributed.^33,34^ Factor rotation was performed through an oblique method (promax, δ = 0), because of potential conceptual correlation among the factors. Examination of scree-plot and parallel analysis^35^ were employed to determine the number of factors, with the latter procedure being performed through a SPSS syntax developed by O’Connor.^36^ Following Floyd & Widaman,^37^ factor loadings above 0.30 were considered relevant. In order to test whether the Resilience Scale has a hierarchical factor structure, a second-order factor analysis was conducted on the four oblique factors in the same manner as previously described. Based on the second-order factor analysis, the Schmid-Leiman orthogonalization procedure^38^ was used to investigate the loading of items in the higher and lower order factors. This procedure was carried out using SPSS syntax codes developed by Wolff & Preising.^39^ Factor loadings equal to or greater than 0.25 are generally considered satisfactory. Cronbach’s alpha values were calculated for the full scale and the extracted factors, for the full sample and split by dementia severity (mild and moderate). Finally, Pearson’s correlations were calculated to establish the relationship between higher- and lower-order factors, as well as the association with clinical variables such as burden among caregivers and cognition, functionality, mood, neuropsychiatric symptoms, and awareness of disease among people with dementia.

## Results

The total Resilience Scale mean score was 140.3 (standard deviation [SD] = 16.28), ranging from 25 to 175, indicating moderate levels of resilience. Cronbach’s alpha was high (α = 0.77), indicating good internal consistency of the scale^38^; internal consistency remained high after dividing the sample according to dementia severity (mild severity: α = 0.78; moderate severity: α = 0.83).^40^ The clinical data of people with AD and their caregivers are shown in Table 1.

**Table 1:** Background variables divided by group

Variable	People with AD (n = 143)	Caregivers (n = 143)
Age	75.2 (9.1)	58.8 (14.3)
Gender, female/male	90/53	118/25
Mini-Mental State Examination	18.8 (4.4)	---
Years of education	8 (4.0)	11.65 (3.16)
Neuropsychiatric Inventory	17.5 (16.7)	---
Pfeffer Functional Activities Questionnaire	16.9 (8.3)	---
Cornell Scale for Depression	7.8 (5.6)	---
Assessment Scale of Psychosocial Impact of the Diagnosis of Dementia	9.4 (5.1)	---
Zarit Burden Interview	---	28.9 (14.5)
Resilience Scale	---	140.3 (16.3)
Clinical Dementia Rating (1/2)	94/49	---

Data presented as mean (standard deviation), unless otherwise specified.

AD = Alzheimer disease.

### Exploratory factor analysis

The KMO analysis revealed a value of 0.63, indicating that the correlation matrix was appropriate for factor analysis. The examination of scree plot and parallel analysis led to a four-factor solution, which accounted for 47% of the variance. Results from the pattern and structure matrix were similar; we report the pattern matrix because results are typically more conservative and not inﬂated by the overlap between factors.^41,42^

Table 2 shows the pattern of rotated factor loads for this four-factor solution. As a whole, the four-factor solution of the Resilience Scale presented a well-defined structure. With the exception of items #2, 4 and 17, which loaded both factors I and II, item #15, which carried the factors II and II, item #23, which carried the factors II and IV, and item #24, which loaded factors II, III and IV, the main charging items are fully highlighted. Items #1, 7, 11, 20 and 22 had no salient charges at any factor (hyperplane items).

**Table 2:** Factor loadings of the 25 Resilience Scale items obtained with maximum likelihood analysis and oblimin rotation

Item #	Item description	Resilience factors			

		I	II	III	IV
3	I am able to depend on myself more than anyone else.	**0.999**	-0.008	0	0
5	I can be on my own if I have to.	**0.574**	0.249	0.01	0.199
2	I usually manage one way or another.	**0.474**	**0.542**	0.093	-0.021
17	My belief in myself gets me through hard times.	**0.39**	**0.444**	0.066	-0.039
9	I feel that I can handle many things at a time.	**0.367**	0.164	0.194	-0.006
4	Keeping interested in things is important to me.	**0.336**	**0.533**	0.165	-0.354
21	My life has meaning.	0.297	**0.726**	-0.229	-0.027
10	I am determined.	0.285	**0.433**	0.289	0.183
15	I keep interested in things.	0.266	**0.603**	**0.46**	-0.243
19	I can usually look at a situation in a number of ways.	0.254	**0.691**	-0.204	0.026
16	I can usually find something to laugh about.	0.236	**0.313**	-0.095	-0.062
8	I am friends with myself.	0.223	**0.46**	0.085	0.072
14	I have self-discipline.	0.217	0.121	**0.309**	0.107
18	In an emergency, I’m someone people can generally rely on.	0.205	**0.449**	0.27	0.247
13	I can get through difficult times because I’ve experienced difficulty before.	0.197	**0.443**	0.281	0.117
1	When I make plans, I follow through with them.	0.192	0.283	0.285	0.221
7	I usually take things in stride.	0.156	0.127	0.01	0.158
6	I feel proud that I have accomplished things in life.	0.156	**0.552**	0.224	-0.108
25	It’s okay if there are people who don’t like me.	0.153	**0.335**	0.164	0.299
23	When I’m in a difficult situation, I can usually find my way out of it.	0.152	**0.486**	0.158	**0.332**
24	I have enough energy to do what I have to do.	0.126	**0.373**	**0.309**	**0.357**
20	Sometimes I make myself do things whether I want to or not.	0.089	0.256	0.15	-0.416
22	I do not dwell on things that I can’t do anything about.	0.072	0.092	0.055	0.119
11	I seldom wonder what the point of it all is.	0.051	-0.095	0.085	0.129
12	I take things one day at a time.	-0.012	0.14	-0.07	**0.306**
Eigenvalue		6.5	1.8	1.6	1.4
Variance (%)		26.1	7.2	6.2	5.7
Cronbach’s alpha		0.85	0.74	0.73	0.71

Factor loadings greater than 0.3 are presented in bold.

The ﬁrst factor was responsible for 26.1% of the variance, with an eigenvalue of 6.5 and an excellent internal consistency (α = 0.81). This factor was composed of six items associated with sense of life and self-sufficiency. The second factor explained 7.2% of the variance, with an eigenvalue of 1.8, and incorporated 15 items associated with resilience levels of perseverance. Factor loadings were moderate to high, and good internal consistency (α = 0.73). The third factor was responsible for 6.2% of the variance, with an eigenvalue of 1.6, and was composed of items related to resilience characteristics such as self-confidence. It had high factor loadings and a good level of internal consistency (α = 0.73). Finally, the fourth factor explained 5.7% of the variance, with an eigenvalue of 1.4, and contained three items associated with equanimity and meaningfulness. It showed high factor loadings and good internal consistency (α = 0.71).

### Hierarchical factor analysis

The second-order factor analysis led to a one-factor solution with an eigenvalue equal to 2.6, which accounted for 54.1% of the variance, and three other factors with eigenvalues smaller than one. This result suggests, indeed, that the Resilience Scale has a hierarchical factor structure, in which the four lower-order factors are loaded on a single higher-order factor. Table 3 shows that the higher-order structure was good and simple. Because the Schmid-Leiman procedure allows the higher-order factor to account for as much of the correlation among the items as possible, while the lower-order factors are reduced to residual factors uncorrelated with each other and with the higher-order factor, factor loadings are generally lower than those observed in the original exploratory factor analysis presented in Table 2. Therefore, factor loadings equal to or greater than 0.25 are generally considered satisfactory.^40^ The higher-order factor accounted for 47.9% of the variance and yielded salient loading on most items. The four lower-order factors explained relatively less of the variance, with the exception of items loading on factor IV (equanimity and meaningfulness), and some items loading on factors II (perseverance) and III (self-confidence). Item loadings across these factors were similar to the pattern observed in Table 2, suggesting the same factor labels, the exception being items from factor I (sense of life and self-sufficiency), which loaded heavily on the higher-order factor. Hyperplane items from the ﬁrst-order analysis loaded better on the higher-order factor. Table 3 shows the hierarchical Resilience Scale structure with loadings for one higher- and four lower-order factors.

**Table 3:** Hierarchical Resilience Scale structure with loadings for one higher- and four lower-order factors

Item #	Higher-order factor	Lower-order factors			

		I	II	III	IV
15	**0.776**	**0.264**	**0.322**	0.056	**0.353**
06	**0.534**	0.188	**0.508**	0.128	**0.251**
18	**0.465**	0.215	0.162	**0.298**	0.064
04	**0.452**	**0.365**	**0.487**	-0.123	**0.481**
14	**0.439**	0.233	0.07	0.105	-0.065
10	**0.385**	**0.34**	**0.301**	-0.103	0.149
02	**0.379**	**0.511**	**0.431**	0.068	**0.357**
01	**0.374**	0.222	0.187	-0.026	-0.035
13	**0.348**	0.199	0.159	**0.262**	**0.293**
05	**0.347**	**0.6**	**0.277**	**0.272**	0.002
08	**0.333**	0.16	0.225	0.094	0.04
24	**0.306**	0.182	0.192	0.163	0.091
19	**0.262**	**0.255**	**0.295**	0.084	0.173
03	0.249	**0.98**	0.202	0.106	0.15
21	0.217	**0.296**	**0.896**	0.109	0.239
09	0.215	**0.416**	0.121	-0.09	**0.258**
23	0.209	**0.241**	**0.34**	0.089	0.192
17	0.181	**0.361**	**0.337**	0.064	**0.38**
25	0.153	0.135	0.016	**0.268**	0.129
22	0.145	0.052	0.01	**0.468**	0.032
16	0.124	0.174	0.247	0.102	0.12
07	0.081	0.144	0.102	**0.374**	0.061
12	0.042	-0.043	0.055	**0.494**	-0.142
20	0.037	0.099	0.163	0.052	**0.737**
11	-0.038	0.029	-0.103	**0.383**	0.075

Factor loadings greater than 0.25 are presented in bold.

### Correlations between factors of the Resilience Scale

As shown in Table 4, the lower-order factors of the Resilience Scale were correlated with each other. In particular, factor IV (equanimity and meaningfulness) showed only weak correlations with other factors, while correlations between factors I (sense of life and self-sufficiency), II (perseverance), and III (self-confidence) were moderate. Likewise, the highest-order factor had moderate to strong correlations with lower-order factors I (sense of life and self-sufficiency), II (perseverance), and III (self-confidence), and a low to moderate correlation with factor IV (equanimity and meaningfulness).

**Table 4:** Correlations between higher-and lower-order Resilience Scale factors

Variable	Higher-order factor	Lower-order factors			

		I	II	III	IV
Higher-order factor	1.00				
Factor I	0.97	1.00			
Factor II	0.81	0.72	1.00		
Factor III	0.71	0.62	0.42	1.00	
Factor IV	0.49	0.35	0.29	0.31	1.00

### Correlations between Resilience Scale factors and clinical variables

Pearson’s correlations were calculated to explore the relationship between total Resilience Scale scores and clinical variables of the people with AD, such as cognition, mood, functional disability, neuropsychiatric symptoms, and awareness of disease, in addition to caregiver burden. To avoid inflation of the family error rate, the p-values were adjusted with the Bonferroni-Hochberg corrections. The results can be seen in Table 5. The Resilience Scale was not correlated with any clinical variable.

**Table 5:** Correlations between Resilience Scale and clinical variables

Variable	Resilience
Pfeffer Functional Activities Questionnaire	*r =* 0.117 p = 0.165
Cornell Scale for Depression	*r =* 0.081 p = 0.334
Assessment Scale of Psychosocial Impact of the Diagnosis of Dementia	*r =* -0.061 p = 0.467
Mini-Mental State Examination	*r =* -0.038 p = 0.65
Neuropsychiatric Inventory	*r =* 0.051 p = 0.548
Clinical Dementia Rating	*r =* -0.002 p = 0.985
Zarit Burden Interview	*r =* 0.058 p = 0.49

## Discussion

This study evaluated the construct validity of the Resilience Scale in caregivers of people with AD. Our findings suggest that the scale is a reliable measure for this population, presenting good internal consistency, with Cronbach’s α = 0.77, which is similar to the result obtained for the Brazilian version by Pesce et al. (0.80).^18^ The coefficients of item-total correlations were within acceptable levels, suggesting adequacy of the items of the Resilience Scale. Our results showed that resilience is a construct with multiple dimensions. The first exploratory factor analysis detected four dimensions related to resilience: sense of life and self-sufficiency (factor I), perseverance (factor II), self-confidence (factor III), and equanimity and meaningfulness (factor IV). Second-order exploratory factor analysis indicated that the four lower-order factors loaded onto a single higher-order factor. Most items yielded expressive loadings in the higher-order factor. In addition, the high-order factor accounted for more than half of the variance.

It is important to highlight that our findings in the exploratory factor analysis differ from the findings obtained with the original scale^1^ and also from those related to the Brazilian validation study whose sample comprised students from public schools.^18^According to the exploratory factor analysis of the original scale,^1^ factor 1 consists of 17 items (#1, 2, 3, 4, 5, 6, 9, 10, 13, 14, 15, 17, 18, 19, 20, 23, and 24) and corresponds to personal competences and the measures self-confidence, independence, determination, invincibility, mastery, ingenuity, and perseverance. Factor 2, consisting of eight items (#7, 8, 11, 12, 16, 21, 22, and 25), corresponds to the acceptance of self and life and measures adaptability, balance, flexibility, and balanced life perspective.

Pesce et al.,^18^ in turn, in the exploratory factor analysis, found three factors that did not distinguish between sense of competence and self-sufficiency. Our findings showed that the four-factor solution of the Resilience Scale presented a well-defined structure. With the exception of some items (#2, 4, 15, 17, 23, and 24) that loaded more than one factor, the main charging items are fully highlighted. Conversely, we may suppose that the five hyperplane items identified (#1, 7, 11, 20, and 22) do not seem to be suitable to our target population due to specific disease characteristics, i.e., people with AD have cognitive deficits and behavioral disturbances that are difficult to be managed by the caregivers.

Furthermore, there are some possible explanations for the differences observed between the exploratory factor analysis performed by Pesce et al.^18^ and our findings. First, we may assume that resilience may vary according to the age of the target population. In the study by Pesce et al.,^18^ the sample comprised students with an age range between 12 and 19 years. Our sample had a mean age of 58.8 (14.3) years, an age range that certainly has an effect on some aspects associated with resilience, such as sense of life or self-confidence. Another explanation is related to the background of the sample and the presence of adverse life events. Wilks & Croom^4^ proposed a model for resilience in which the abilities and characteristics of the individual (competence) are related to the demands and resources of the physical or social environment.^10^ Caregivers of people with AD may have personal aspects that are influenced by the disease, such as lack of social interactions, financial difficulties, frustration, anxiety, reduction of leisure activities, and concerns about the future.^10^ Therefore, future studies should explore the psychometric properties of the Resilience Scale by comparing samples with similar backgrounds.

It is worth mentioning that the mean score obtained in the Resilience Scale was 140.3 points (SD = 16.28), showing that caregivers had moderate to high resilience. Correlations between Resilience Scale scores and clinical aspects of AD (neuropsychiatric symptoms, functionality, mood, awareness of disease, and burden) were not statistically significant. Therefore, we assume that resilience may not be related to the clinical characteristics of people with AD.^43^ The subjective assessment of caregivers’ individual characteristics may be a determinant of the situation, i.e., the perception, interpretation, and sense attributed to the stressor event may be or not classified as a stressful condition.^18^ However, the lack of relationship between resilience and the clinical aspects of AD is derived from a correlation analysis that does not allow to infer any causality.

### Limitations

The present study has some limitations that should be considered. Firstly, we recruited caregivers from only one center of treatment for people with dementia. Secondly, this was a cross-sectional study focused on caregivers of people with AD, and the findings cannot be generalized to caregivers of people with other types of dementia. In addition, we did not study the concurrent validity of the Resilience Scale as compared to any other resilience assessment. Finally, we have not assessed the history of neurological and psychiatry disorders in caregivers, which would allow to investigate their impact on caregivers’ resilience.

## Conclusion

To the best of our knowledge, there is no standardized resilience measure in Brazil other than the Resilience Scale, used to investigate caregivers of people with AD. The factor structure of the scale obtained through exploratory factor analysis provides strong evidence for construct validity, indicating that the Resilience Scale is a reliable measure of resilience in AD. The multidimensional nature of the resilience concept was confirmed by the factor analysis, which identified four dimensions of resilience related to sense of life and self-sufficiency, perseverance, self-confidence, and equanimity and meaningfulness. Investigating the underlying mechanisms associated with the differences and degrees of resilience will allow to enhance the understanding of risk factors associated with resilience and to improve coping strategies and self-efficacy among caregivers of people with AD.

## References

[1] 1. Wagnild GM, Young HM. Development and psychometric evaluation of resilience scale. J Nurs Meas 1993;1:165-78. 7850498

[2] 2. Dias R, Santos RL, de Sousa MF, Nogueira MM, Torres B, Belfort T, et al. Resilience of caregivers of people with dementia: a systematic review of biological and psychosocial determinants. Trends Psychiatry Psychother. 2015;37:12-9. 10.1590/2237-6089-2014-0032 25860562

[3] 3. Windle G, Bennett KM. Caring relationships: how to promote resilience in challenging times. In: Ungar M, ed. The social ecology of resilience: a handbook of theory and practice. New York: Springer; 2011. p. 219-31.

[4] 4. Wilks SE, Croom B. Perceived stress and resilience in Alzheimer’s disease caregivers: testing moderation and mediation models of social support. Aging Ment Health. 2008;12:357-65. 10.1080/13607860801933323 18728949

[5] 5. Han S, Chi NC, Han C, Oliver DP, Washington K, Demiris G, et al. Adapting the resilience framework for family caregivers of hospice patients with dementia. Am J Alzheimers Dis Other Demen. 2019;34:399-411. 10.1177/1533317519862095 PMC7179812 31364381

[6] 6. Masten AS, Tellegen A. Resilience in develop mental psychopathology: contributions of the project competence longitudinal study. Dev Psychopathol. 2012;24:345-61. 10.1017/S095457941200003X 22559118

[7] 7. Bekhet AK. Effects of positive cognitions and resourcefulness on caregiver burden among caregivers of persons with dementia. Int J Ment Health Nurs. 2013;22:340-6. 10.1111/j.1447-0349.2012.00877.x 23009397

[8] 8. Contador I, Fernández-Calvo B, Palenzuela DL, Miguéis S, Ramos F. Prediction of burden in family caregivers of patients with dementia: a perspective of optimism based on generalized expectancies of control. Aging Ment Health. 2012;16:675-82. 10.1080/13607863.2012.684666 22746193

[9] 9. Dourado MC, de Sousa MF, Santos RL, Simões Neto JP, Nogueira ML, Belfort TT, et al. Quality of life in mild dementia: patterns of change in self and caregiver ratings over time. Braz J Psychiatry. 2016;38:294-300. 10.1590/1516-4446-2014-1642 PMC7111349 26785107

[10] 10. da Rosa RD, Simões-Neto JP, Santos RL, Torres B, Baptista MA, Kimura NR, et al. Caregivers’ resilience in mild and moderate Alzheimer’s disease. Aging Ment Health. 2020;24:2:250-8. 10.1080/13607863.2018.1533520 30499333

[11] 11. O’Rourke N, Kupferschmidt AL, Claxton A, Smith JZ, Chappell N, Beattie BL. Psychological resilience predicts depressive symptoms among spouses of persons with Alzheimer disease over time. Aging Ment Health. 2010;14:984-93. 10.1080/13607863.2010.501063 21069604

[12] 12. Gaugler JE, Kane RL, Newcomer R. Resilience and transitions from dementia caregiving. J Gerontol B Psychol Sci Sco Sci. 2007;62:P38-44. 10.1093/geronb/62.1.p38 17284556

[13] 13. Fernández-Lansac V, Crespo López M, Cáceres R, Rodríguez- Poyo M. Resilience in caregivers of patients with dementia: A preliminary study. Rev Esp Geriatr Gerontol. 2012;47:102-9. 10.1016/j.regg.2011.11.004 22579610

[14] 14. Bull MJ. Strategies for sustaining self used by family caregivers for older adults with dementia. J Holist Nurs. 2014;32:127-35. 10.1177/0898010113509724 24227181

[15] 15. Shuter P, Beattie E, Edwards H. An exploratory study of grief and health-related quality of life for caregivers of people with dementia. Am J Alzheimers Dis Other Demen. 2013;29:379-85. 10.1177/1533317513517034 PMC10852965 24381138

[16] 16. Clay OJ, Roth DL, Wadley VG, Haley WE. Changes in social support and their impact on psychosocial outcome over a 5-year period for African American and white dementia caregivers. Int J Geriatr Psychiatry. 2008;23:857-62. 10.1002/gps.1996 18338341

[17] 17. Cowan PA, Cowan CP, Schulz MS. Thinking about risk and resilience in families. In: Hethering EM, Blecheman EA, editors. Stress, coping and resilience in children and families. New Jersey: Lawrence Erlbaum; 1996. p. 1-38.

[18] 18. Pesce RP, Assis SG, Avanci JQ, Santos NC, Malaquias JV, Carvalhaes R. [Cross-cultural adaptation, reliability and validity of the resilience scale]. Cad Saude Publica. 2005;21:436-48. 10.1590/s0102-311x2005000200010 15905906

[19] 19. American Psychiatric Association. Diagnostic and Statistical Manual of Mental Disorders, Fifth Edition (DSM-5). Arlington: American Psychiatric Publishing; 2013.

[20] 20. Treiblmaier H, Filzmoser P. Exploratory factor analysis revisited: how robust methods support the detection of hidden multivariate data structures in IS research. Inf Manag. 2010;47:197-207.

[21] 21. Morris J. The CDR: current version and scoring rules. Neurol. 1993;43:2412-4. 10.1212/wnl.43.11.2412-a 8232972

[22] 22. Folstein MF, Folstein SE, McHugh PR. Mini-mental state: a practical method for grading the cognitive state of patients for the clinician. J Psychiatr Res. 1975;12:189-98. 10.1016/0022-3956(75)90026-6 1202204

[23] 23. Dourado M, Marinho V, Soares C, Engelhardt E, Laks J. Awareness of disease in dementia: development of a multidimensional rating scale. Dement Neuropsychol. 2010;1:74-80. 10.1590/S1980-57642008DN10100012 PMC5619387 29213371

[24] 24. Alexopoulos GS, Abrams RC, Young RC, Shamoian CA. Cornell scale for depression in dementia. Biol Psychiatry. 1998;23:271-84. 10.1016/0006-3223(88)90038-8 3337862

[25] 25. Carthery-Goulart MT, Areza-Fegyveres R, Schultz RR, Okamoto I, Caramelli P, Bertolucci PH, et al. [Brazilian version of the Cornell depression scale in dementia]. Arq Neuropsiquiatr. 2007;65:912-5. 10.1590/s0004-282x2007000500037 17952308

[26] 26. Scazufca M. Brazilian version of the Burden Interview scale for the assessment of burden of care in carers of people with mental illnesses. Braz J Psychiatry. 2002;24:12-7.

[27] 27. Camozzato AL, Kochhann R, Simeoni C, Konrath CA, Pedro Franz A, Carvalho A, et al. Reliability of the Brazilian Portuguese version of the Neuropsychiatric Inventory (NPI) for patients with Alzheimer’s disease and their caregivers. Int Psychogeriatr. 2008;20:383-93. 10.1017/S1041610207006254 18257965

[28] 28. Maia AL, Godinho C, Ferreira ED, Almeida ED, Schuh V, Kaye, et al. Application of the Brazilian version of the CDR scale in samples of dementia patients. Arq Neuropsiquiatr. 2006;64:485-9. 10.1590/s0004-282x2006000300025 16917624

[29] 29. Nitrini R, Caramelli P, Bottino CM, Damasceno BP, Brucki SM, Anghinah R. Diagnóstico de doença de Alzheimer no Brasil: avaliação cognitiva e funcional. Arq Neuropsiquiatr. 2005;63:720-7. 10.1590/s0004-282x2005000400034 16172733

[30] 30. Revelle W. How to: use the psych package for factor analysis and data reduction [Internet]. 2016 [cited 2021 Feb 5]. cran.r-project.org/web/packages/psychTools/vignettes/factor.pdf

[31] 31. Tabachnick BG, Fidell LS. Using multivariate statistics. Harper Collins: Allyn & Bacon; 2001.

[32] 32. Costello AB, Osborne JW. Best practices in exploratory factor analysis: four recommendations for getting the most from your analysis. Pract Assess Res Eval. 2005;10:1-9.

[33] 33. Firth D. Bias reduction of maximum likelihood estimates. Biometrika. 1993;80:27-38.

[34] 34. Olsson U. Maximum likelihood estimation of the polychoric correlation coefﬁcient. Psychometrika. 1979;44:443-60.

[35] 35. Hayton JC, Allen DG, Scarpello V. Factor retention decisions in exploratory factor analysis: a tutorial on parallel analysis. Organ Res Methods. 2004;7:191-205.

[36] 36. O’Connor BP. SPSS and SAS programs for determining the number of components using parallel analysis and Velicer’s MAP test. Behav Res Methods Instrum Comput. 2000;32:396-402. 10.3758/bf03200807 11029811

[37] 37. Floyd FJ, Widaman KF. Factor analysis in the development and reﬁnement of clinical assessment instruments. Psychol Assess. 1995;7:286-99.

[38] 38. Schmid J, Leiman JM. The development of hierarchical factor solutions. Psychometrika. 1957;22:53-61.

[39] 39. Wolff HG, Preising K. Exploring item and higher order factor structure with the Schmid-Leiman solution: syntax codes for SPSS and SAS. Behav Res Methods. 2005;37:48-58. 10.3758/bf03206397 16097343

[40] 40. George D, Mallery P. SPSS for Windows step by step: a simple guide and reference. 11.0 update. Boston: Allyn & Bacon; 2003.

[41] 41. Brown T. Conﬁrmatory factor analysis for applied research. New York: Guilford; 2006.

[42] 42. Hatcher L. A step-by-step approach to using the SAS System for factor analysis and structural equation modeling. Cary: SAS Institute Inc,; 1994.

[43] 43. Dias R, Simões-Neto JP, Santos RL, Sousa MFB, Baptista MA, Lacerda IB, et al. Caregivers’ resilience is independent from the clinical symptoms of dementia. Arq Neuropsiquiatr. 2016;74:967-73. 10.1590/0004-282X20160162 27991993

